# Phylogenetic and Comparative Genomics Study of Papilionidae Based on Mitochondrial Genomes

**DOI:** 10.3390/genes15070964

**Published:** 2024-07-22

**Authors:** Zhen-Tian Yan, Xiao-Ya Tang, Dong Yang, Zhen-Huai Fan, Si-Te Luo, Bin Chen

**Affiliations:** 1 Chongqing Key Laboratory of Vector Control and Utilization, Institute of Entomology and Molecular Biology, College of Life Sciences, Chongqing Normal University, Chongqing 401331, China; 2 School of Life Sciences, Xiamen University, Xiamen 361102, China

**Keywords:** *Byasa confusa*, papilionidae, mitogenomes, phylogenetic analysis

## Abstract

Most species of Papilionidae are large and beautiful ornamental butterflies. They are recognized as model organisms in ecology, evolutionary biology, genetics, and conservation biology but present numerous unresolved phylogenetic problems. Complete mitochondrial genomes (mitogenomes) have been widely used in phylogenetic studies of butterflies, but mitogenome knowledge within the family Papilionidae is limited, and its phylogeny is far from resolved. In this study, we first report the mitogenome of *Byasa confusa* from the subfamily Papilioninae of Papilionidae. The mitogenome of *B. confusa* is 15,135 bp in length and contains 13 protein-coding genes, 22 transfer RNA genes, 2 ribosomal RNA genes, and an AT-rich control region (CR), closely mirroring the genomic structure observed in related butterfly species. Comparative analysis of 77 Papilionidae mitogenomes shows gene composition and order to be identical to that of an ancestral insect, and the AT bias, Ka/Ks, and relative synonymous codon usage (RSCU) are all consistent with that of other reported butterfly mitogenomes. We conducted phylogenetic analyses using maximum-likelihood (ML) and Bayesian-inference (BI) methods, with 77 Papilionidae species as ingroups and two species of Nymphalidae and Lycaenidae as outgroups. The phylogenetic analysis indicated that *B. confusa* were clustered within *Byasa*. The phylogenetic trees show the monophyly of the subfamily Papilioninae and the tribes Leptocircini, Papilionini, and Troidini. The data supported the following relationships in tribe level on Papilioninae: (((Troidini + Papilionini) + Teinopalpini) + Leptocircini). The divergence time analysis suggests that Papilionidae originated in the late Creataceous. Overall, utilizing the largest number of Papilionidae mitogenomes sequenced to date, with the current first exploration in a phylogenetic analysis on Papilionidae (including four subfamilies), this study comprehensively reveals the mitogenome characteristics and mitogenome-based phylogeny, providing information for further studies on the mitogenome, phylogeny, evolution, and taxonomic revision of the Papilionidae family.

## 1. Introduction

The Papilionidae family encompasses four subfamilies (Papilioninae, Zerynthinae, Baroniinae, and Parnassinae), comprising approximately 570 species worldwide, with the majority inhabiting tropical and subtropical regions [1,2]. Mitogenome data have been pivotal in inferring and analyzing the phylogenetic relationships of butterflies, serving as reliable molecular markers due to maternal inheritance, stable gene composition, relatively conserved gene sequences, and low recombination rates [3,4]. Butterfly research often utilizes mitochondrial markers like *COI*, *COII*, and *16S rRNA* for studies in molecular systematics, evolution, population genetics, and phylogenetics [5,6]. However, complete mitochondrial genomes for Papilionidae remain scarce, hindering a comprehensive understanding of phylogenetic relationships within the family. Uncertainties persist regarding the classification of Zerynthinae and the taxonomic status of Parnassinae [7]. To date, complete mitochondrial genome sequences are unavailable for most *Byasa* species, with only *Byasa alcinous* [8] and *Byasa hedistus* [9] having been reported. Lack of genomic data hinders our ability to elucidate the phylogenetic relationships within the Papilionidae family.

*Byasa confusa* (Rothschild, 1896), a member of the Papilionidae family’s Papilioninae subfamily, belongs to the genus *Byasa* and is primarily distributed in China, with occurrences also documented in Japan, Korea, and Vietnam [10,11]. Renowned as an essential ornamental butterfly, previous research on this species has predominantly concentrated on morphological characteristics, artificial rearing, and behavioral observations [7,8,11,12].

In this study, we present the first-time sequencing and analysis of the complete mitochondrial genome of *B. confusa*. Our analysis incorporated a total of 77 mitochondrial genomes, including one newly sequenced mitogenome, 76 previously published mitochondrial genomes from various Papilionidae species (encompassing 4 subfamilies and 31 genera), along with two outgroups sourced from the Nymphalidae and Lycaenidae families. Not only did we provide a preliminary comparative analysis of their genetic composition and structural features but we also constructed phylogenetic relationships and discussed the evolution among numerous significant subfamilies and tribes within Papilionidae.

## 2. Materials and Methods

### 2.1. Sample Collection and Identification

**Figure 1:**
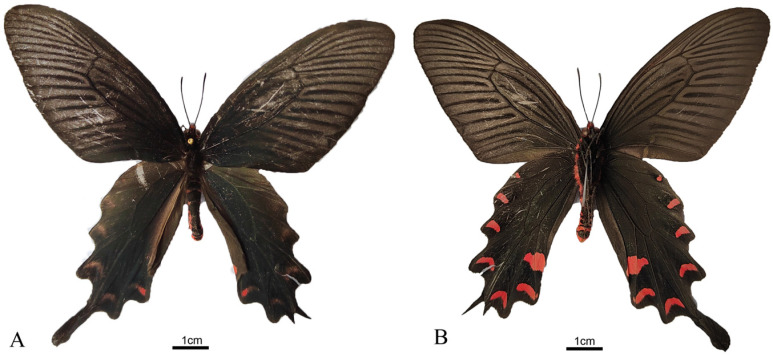
Images of a male adult specimen of *B. confusa*: (**A**) dorsal; (**B**) ventral. (photographed by Zhentian Yan).

Adults of *B. confusa* (Figure 1) were collected by sweep nets in Xingtian Village of Dongan Town (109°11′2″ E, 31°42′46″ N), Chengkou District in Chongqing, China. The specimens were morphologically identified (Figure 1) and confirmed by referring to previous studies [11]. Voucher specimens were deposited at Chongqing Normal University (CQNU) (accession number 20170700203, Zhentian Yan, 20132148@cqnu.edu.cn).

### 2.2. Sequencing and Mitogenome Assembly

Genomic DNA extraction from male legs was performed using a TIANamp Genomic DNA Kit (TIANGEN, Beijing, China) [13]. Subsequently, the DNA library was prepared according to the manufacturer’s guidelines, employing the Illumina TruSeq™ DNA Sample Preparation Kit (Illumina, San Diego, CA, USA). The constructed library was then loaded onto an Illumina Novaseq 6000 platform for PE 2 × 150 bp sequencing, which was performed by Novogene (Beijing, China). Quality control and filtering of low-quality reads were conducted using the NGS QC toolkit [14]. The clean data obtained were utilized for assembling the complete mitogenome through the GetOrganelle pipeline, using the “animal_mt” parameter [15]. 

### 2.3. Mitogenome Annotation and Characteristics Analysis

Assembled mitochondrial sequences were annotated using an MitoZ (v3.4) annotation module [16]. The annotated genomes have been deposited in GenBank under the accession number PP375289. Initial determination of gene boundaries was conducted through the MITOS web server to identify the locations of each gene [17]. Comparison with Papilionidae mitogenomes, available on GenBank, allowed the identification of 13 protein-coding genes (PCGs) and 2 ribosomal RNA (rRNA) genes. Genomes were visualized using CGView [18].

Amino acid composition, nucleotide content, and relative synonymous codon usage (RSCU) were examined using MEGA software (v6.06) and PhyloSuite (v1.2.3) [19,20]. Codon usage bias for a broader sample of 77 Papilionidae species was analyzed using the pheatmap package in R (v4.2.2), implemented in Hiplot Pro (https://hiplot.com.cn/, accessed on 6 June 2024) [21]. Nucleotide compositional bias was assessed by AT/GC skew. 

Rates of synonymous (Ks) and non-synonymous (Ka) mutations were calculated using DnaSP (v5.10.01) [21]. Nucleotide diversity (Pi) was calculated with a 100-bp sliding window approach in DnaSP (v5.10.01). Box plots and 3D scatter plots illustrating AT skew, GC skew, and AT percentage were created in Origin Pro (v9.0) [22].

### 2.4. Phylogenetic Analysis

Phylogenetic relationships among 77 Papilionidae species were investigated, comprising one newly sequenced species from this study and 76 known species (Table 1). The mitochondrial genome database utilized for analysis included protein-coding genes with all codon positions (PCG123), along with two ribosomal RNA genes (*12S rRNA* and *16S rRNA*). Bayesian-inference (BI) and maximum-likelihood (ML) methods were employed for phylogenetic inference. *Vanessa indica* (NC_038157) (Lepidoptera: Nymphalidae) and *Cupido argiades* (KC310728) (Lepidoptera: Lycaenidae) sequences were employed as outgroups. Each mitochondrial gene was aligned separately using MAFFT (v7.388) with default settings [23], with ambiguous regions subsequently removed using Gblocks [24]. Phylogenetic analyses were conducted utilizing PhyloSuite (v1.2.2) [21], with optimal partition schemes and nucleotide substitution models determined via PartitionFinder 2.0 based on the Bayesian information criterion (BIC) [25,26]. BI analysis was executed using MrBayes 3.2.6, employing 2,000,000 generations and 4 chains, with sampling conducted every 1000 generations [25]. A consensus tree was generated after discarding the initial 25% of trees as burn-in, and posterior probabilities (PPs) were calculated. Model selection was performed using the Akaike information criterion (AIC) in ModelFinder [27]. Subsequently, the ML phylogenetic tree was reconstructed using IQ-TREE (v 2.1.2) with 1000 ultrafast bootstraps, employing the GTR + F + R6 model [28].

### 2.5. Divergence Time Estimation

To calibrate the phylogenetic tree using fossil-based age constraints, we included 79 mitogenomes. A fossil age of 108 million years ago (Mya), sourced from timetree.org [29], was utilized. Lognormal priors and fixed hard minimum ages were applied to these fossil calibrations. A dataset containing concatenated protein-coding genes (PCGs) from the 79 mitogenomes was aligned using Clustal. Divergence time estimation was performed using BEAST v2.7.7 [30], with XML file creation done using BEAUTi v2.7.7. BEAUTi was configured with a relaxed uncorrelated lognormal clock model, Yule process speciation model, and GTR + γ site model. Markov chain Monte Carlo (MCMC) chains ran for 500 million generations, sampling trees every 10,000 generations. Tree summarization utilized TreeAnnotator v2.6.7, and visualization was performed using FigTree v1.4.4 [31] to display tree topology, posterior probability support values, and node ages.

**Table 1:** Information on the mitochondrial genome used for the phylogenetic analysis of Papilionidae in this study.

Subfamily	Tribe	Genus	Species	GenBank Accession No.	Reference
Papilioninae	Leptocircini	*Lamproptera*	*Lamproptera curius*	NC_023953	[32]
			*Lamproptera meges*	NC_037867	Unpublished
		*Graphium*	*Graphium chironides*	NC_026910	[33]
			*Graphium sarpedon*	NC_070049	Unpublished
			*Graphium confucius*	OK136253	Unpublished
			*Graphium eurous*	MW549198	Unpublished
			*Graphium mullah*	MW549197	Unpublished
			*Graphium parus*	MT198821	[34]
			*Graphium doson*	MK144328	[35]
			*Graphium leechi*	NC_034837	Unpublished
			*Graphium timur*	NC_024098	[36]
		*Paranticopsis*	*Paranticopsis xenocles*	MZ394042	[37]
		*Pathysa*	*Pathysa antiphates*	NC_069979	Unpublished
		*Iphiclides*	*Iphiclides podalirius*	MK507891	[38]
		*Protographium*	*Protographium marcellus*	MK507890	[38]
		*Protesilaus*	*Protesilaus protesilaus*	LT999984	Unpublished
		*Mimoides*	*Mimoides lysithous*	NC_037871	Unpublished
	Teinopalpini	*Teinopalpus*	*Teinopalpus aureus*	OL449692	[39]
			*Teinopalpus imperialis*	NC_027108	[40]
		*Meandrusa*	*Meandrusa sciron*	LS975123	Unpublished
	Papilionini	*Papilio*	*Papilio natewa*	NC_069293	Unpublished
			*Papilio nephelus*	MZ353681	[41]
			*Papilio alcmenor*	NC_071889	Unpublished
			*Papilio bianor*	KC433409	[42]
			*Papilio helenus*	ON653407	Unpublished
			*Papilio thoas*	NC_059755	Unpublished
			*Papilio polytes*	MZ188895	Unpublished
			*Papilio rex*	NC_034356	Unpublished
			*Papilio slateri*	NC_037874	Unpublished
			*Papilio maackii*	KC433408	[43]
			*Papilio syfanius*	NC_023978	[44]
			*Papilio glaucus*	KR822739	[45]
			*Papilio epycides*	MZ501807	[43]
			*Papilio macilentus*	NC_067884	Unpublished
			*Papilio dialis*	NC_066472	Unpublished
			*Papilio paris*	NC_053770	[46]
		*Agehana*	*Papilio elwesi*	OK052950	[47]
			*Papilio maraho*	NC_014055	[48]
	Troidini	*Euryades*	*Euryades corethrus*	NC_037862	Unpublished
		*Cressida*	*Cressida cressida*	MK507889	[38]
		*Pachliopta*	*Pachliopta aristolochiae*	NC_034280	Unpublished
		*Byasa*	*Byasa alcinous*	LT999969	Unpublished
			*Byasa confusa*	PP375289	This study
		*Atrophaneura*	*Atrophaneura dixoni*	LT999977	Unpublished
		*Parides*	*Parides photinus*	LS974638	Unpublished
		*Pharmacophagus*	*Pharmacophagus antenor*	LS975119	Unpublished
		*Troides*	*Troides aeacus*	NC_060569	Unpublished
		*Losaria*	*Losaria neptunus*	NC_037868	Unpublished
		*Trogonoptera*	*Trogonoptera brookiana*	NC_037875	Unpublished
		*Ornithoptera*	*Ornithoptera priamus*	NC_037870	Unpublished
			*Ornithoptera alexandrae*	NC_073567	Unpublished
			*Ornithoptera richmondia*	NC_037869	Unpublished
Zerynthiinae	Zerynthiini	*Zerynthia*	*Zerynthia polyxena*	MK507888	[38]
		*Luehdorfia*	*Luehdorfia chinensis*	KU360130	[49]
			*Luehdorfia taibai*	NC_023938	[50]
			*Luehdorfia puziloi*	OP936022	[51]
	/	*Allancastria*	*Allancastria cerisyi*	LS974636	Unpublished
		*Bhutanitis*	*Bhutanitis thaidina*	OP894929	[51]
			*Bhutanitis mansfieldi*	NC_037863	Unpublished
Baroniinae	Baroniini	*Baronia*	*Baronia brevicornis*	LT999970	Unpublished
Parnassiinae	Parnassiini	*Parnassius*	*Parnassius bremeri*	NC_014053	[13]
			*Parnassius honrathi*	NC_072292	[51]
			*Parnassius nomion*	OP989703	[51]
			*Parnassius apollo*	OP850800	[51]
			*Parnassius mercurius*	NC_047306	[52]
			*Parnassius orleans*	OP850799	[51]
			*Parnassius szechenyii*	OP850798	[51]
			*Parnassius patricius*	NC_072294	[51]
			*Parnassius schultei*	NC_072290	[51]
			*Parnassius imperator*	NC_072289	[51]
			*Parnassius loxias*	NC_072288	[51]
			*Parnassius hide*	NC_072287	[51]
			*Parnassius simo*	NC_072286	[51]
			*Parnassius choui*	KY072797	Unpublished
			*Parnassius cephalus*	NC_026457	[53]
	/	*Hypermnestra*	*Hypermnestra helios*	LS975127	Unpublished
		*Archon*	*Archon apollinus*	LT999971	Unpublished
Nymphalinae		*Vanessa*	*Vanessa indica*	NC_038157	[54]
Polyommatinae		*Cupido*	*Cupido argiades*	KC310728	[55]

## 3. Results

### 3.1. Genome Organization and Nucleotide Composition

**Figure 2:**
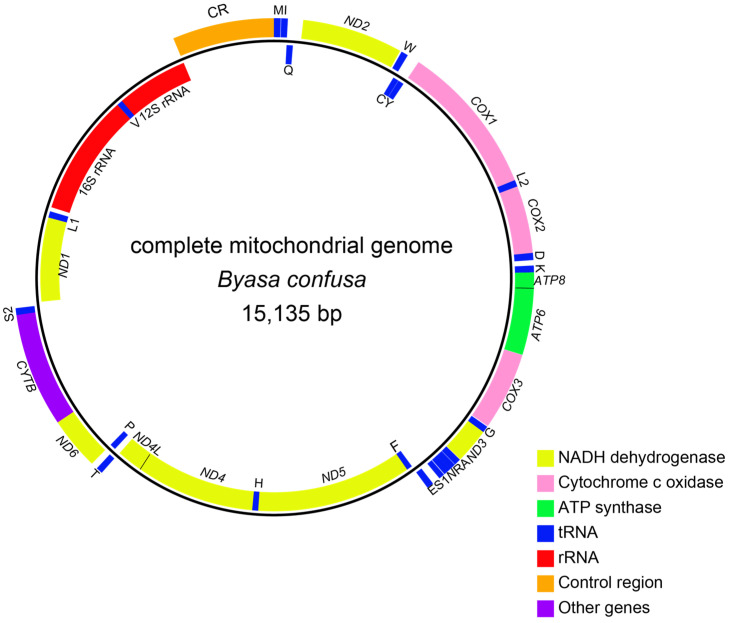
Complete mitogenomic structures of *B. confusa*. The genes on the outer loop are on the J-strand, and the genes on the inner loop are on the N-strand. Different colors indicate different gene families.

The complete mitogenome of *B. confusa* spans a length of 15,135 bp, with an average read coverage of 239-fold (Figure 2). The genome comprises 37 typical mitogenome genes, including 13 PCGs, 22 tRNA genes, and 2 rRNA genes, alongside a non-coding region known as the A + T rich region or control region. The J-strand, the majority coding strand, houses 9 PCGs and 14 transfer RNAs (tRNAs). The remaining genes reside on the N-strand, the minority strand (Figure 2, Table 2).

**Figure 3:**
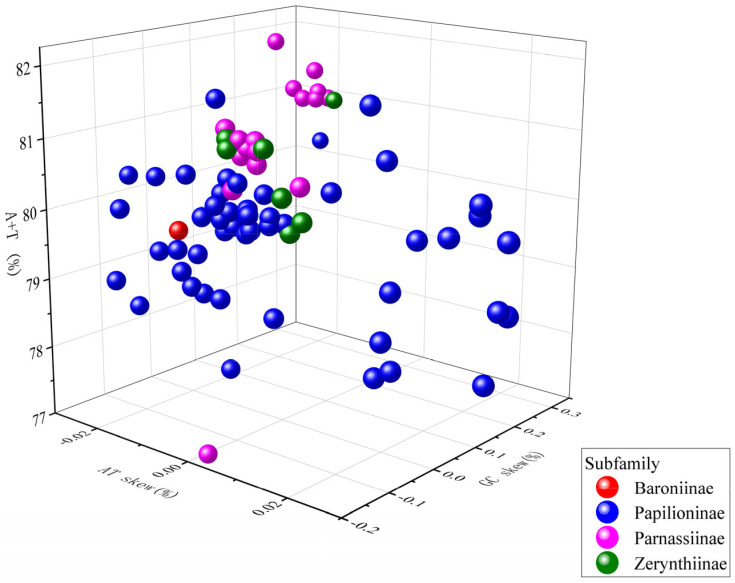
Three-dimensional scatter plot of the AT–skew, GC–skew, and AT% of 77 mitochondrial genome sequences of the family Papilionidae.

In the present study, the mitogenomes of 77 species (including the newly sequenced ones) in Papilionidae were included in our phylogenetic analyses. All mitogenomes exhibit a significant AT bias, with AT content ranging from 77.2% (*Parnassius bremeri*) to 82.2% (*Bhutanitis mansfieldi*), and an average positive AT-skew of 0.0132 with a negative GC-skew of −0.1587 (Figure 3, Table S1). Notably, Parnassinae and Zerynthiinae are more compact relative to Papilioninae in the three-dimensional distribution.

Consistent with other Papilionidae species [31,34,38,56], the mitochondrial gene arrangement of *B. confusa* was observed. The overall nucleotide composition comprises 38.2% A, 42.8% T, 11.6% C, and 7.5% G. The mitochondrial genome exhibits 12 gene overlaps (ranging from 1 to 25 bp). Ribosomal RNAs demonstrate conservation typical of other insects, with the *16S rRNA* spanning 1359 bp, situated between *tRNA^Leu^* and *tRNA^Val^*, and exhibiting an AT content of 84.4%. The *12S rRNA*, flanked by *tRNA^Val^* and the control region, spans 804 bp, with an AT content of 85.1%. Among the 22 interspersed tRNA genes, totaling 1444 bp in length, the AT content is 81.7%, with *tRNA^Glu^* displaying the highest (92.7%) and *tRNA^Lys^* the lowest (70.0%) AT content (Table 3). Consistent with other Papilionidae, the anticodons of the 22 tRNA genes remain identical. The control region spans 303 bp, boasting an A + T content of 95.1% (Table 3), commonly recognized as the origin of DNA replication and exhibiting considerable sequence divergence from other Papilionidae.

### 3.2. Protein-Coding Genes and Codon Usage

As can be seen from Table 2, most protein-coding genes commence with the standard ATN (ATG, ATT, ATA, and ATC) as start codons. Termination codons for the 13 protein-coding genes predominantly consist of TAA, TAG, or T. Specifically, nine protein-coding genes (*ND2*, *COI*, *ATP8*, *ATP6*, *COII*, *ND5*, *ND4L*, *Cytb*, and *ND1*) conclude with the complete termination codon TAA, while *COII* and *ND4* terminate with the incomplete stop codon T. Additionally, *ND3* employs TAG, and *ND6* uses TAT as stop codons (Table 2). The incomplete stop codon T may undergo post-transcriptional polyadenylation to complete as TAA.

**Figure 4:**
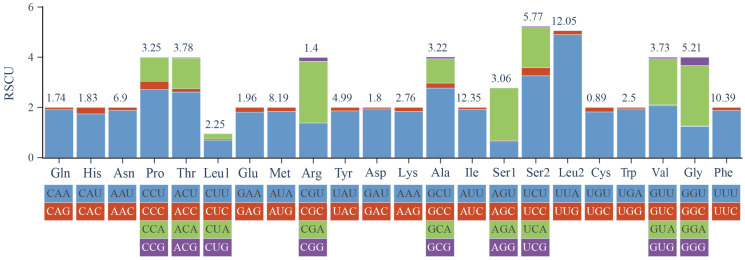
Relative synonymous codon usage of the mitochondrial genome of *B. confusa*.

**Figure 5:**
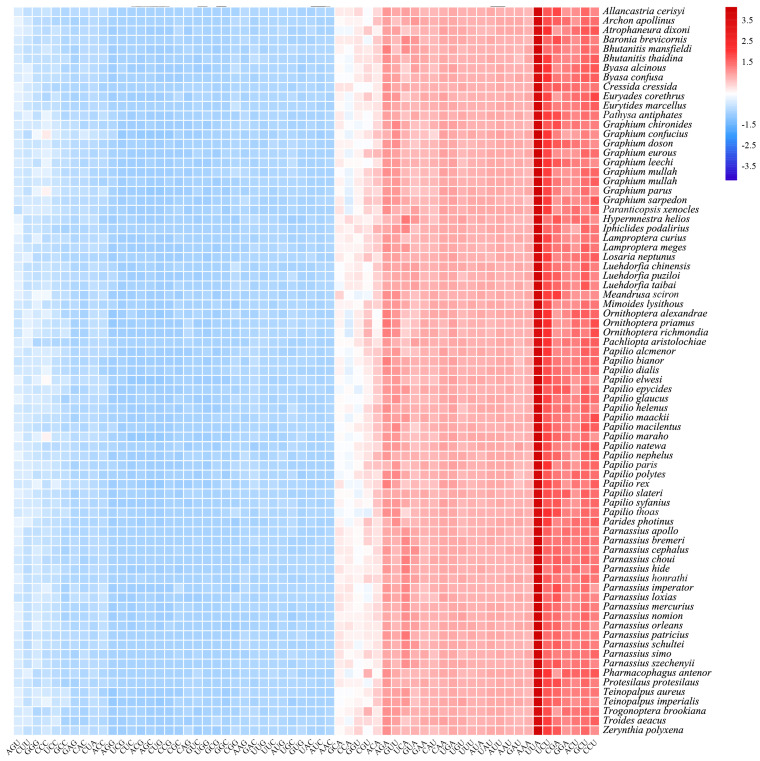
The relative synonymous codon usage (RSCU) of 13 PCGs in the mitogenomes of Papilionidae. The *x*– and *y*–axis represent the codon type and species name, respectively. The legend in the upper right corner represents the usage frequency of synonymous codons.

Analysis of relative synonymous codon usage (RSCU) in the *B. confusa* mitogenome (Figure 4) revealed a preference for codons encoding phenylalanine (Phe), isoleucine (Ile), and leucine (Leu). Conversely, codons for cysteine (Cys) and arginine (Arg) were used the least frequently. This bias aligns with the high Adenine (A) and Thymine (T) content observed in the protein-coding genes. Further supporting this trend, RSCU analysis across Papilionidae (Figure 5) identified the five most common codons (UUA, AUU, CCU, GCU, AUA, and ACU) as A or U-rich, highlighting a strong bias towards AT nucleotides.

### 3.3. Analysis of Nucleotide Diversity and Evolutionary Rate in the Family Papilionidae

**Figure 6:**
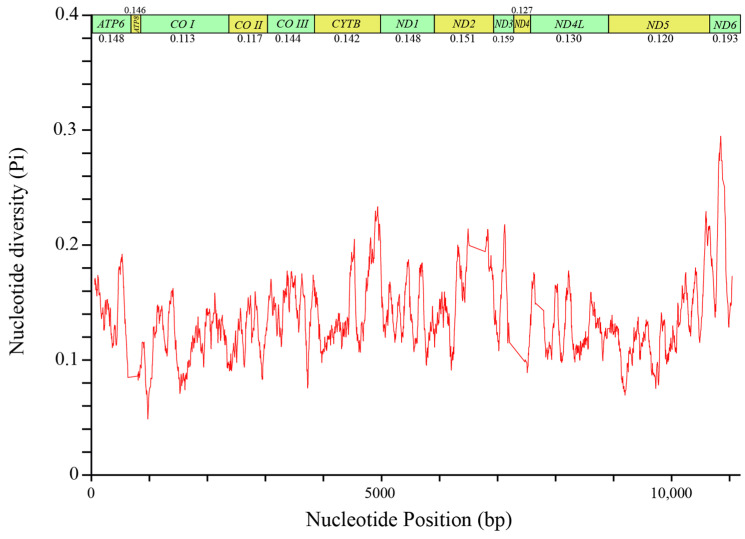
Nucleotide diversity (Pi) was estimated in the mitochondrial genomes of 77 species in the family Papilionidae. The size of the sliding window is 100 bp, with each step being 5 bp. The bars above the graph represent protein-coding genes (PCGs).

**Figure 7:**
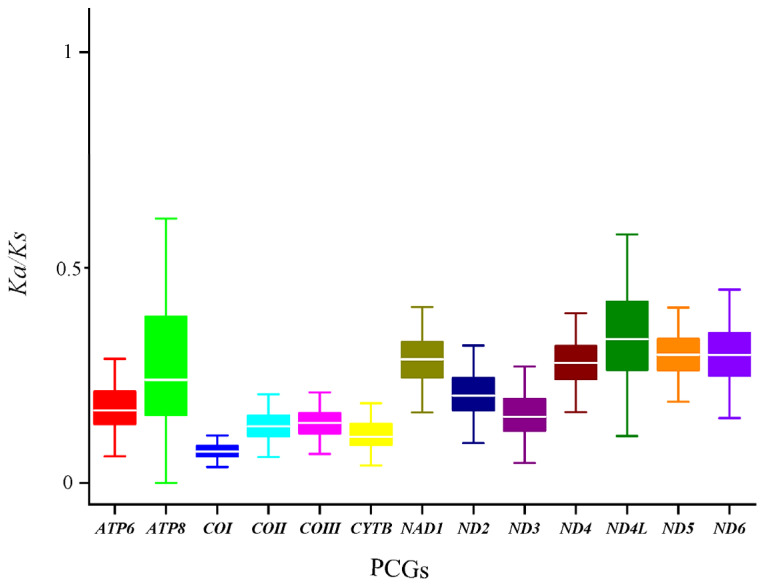
Box plot of Ka/Ks from 13 PCGs of 77 Papilionidae mitogenomes. Ka, non-synonymous mutation rate; Ks, synonymous mutation rate; Ka/Ks, the ratio of non-synonymous mutation rate to synonymous mutation rate.

Analysis of nucleotide diversity within the 13 PCGs of *B. confusa* revealed variation, ranging from 0.113 (*COI*) to 0.193 (*NAD6*) (Figure 6). The *NAD6* gene exhibited the highest diversity, followed by *NAD3*, *NAD2*, and *ATP6*. Conversely, *COI*, *COII*, and *NAD5* displayed lower diversity, suggesting they are more conserved (Figure 6). These findings are further supported by evolutionary rate analysis (Figure 7).

By comparing and analyzing the sizes of Ka, Ks, and Ka/Ks values of the 13 PCGs of the known mitochondrial genomes of 77 butterfly species from Papilionidae, although there are some differences in the Ka and Ks values of the 13 PCGs, it can be seen that all of them have a Ka/Ks value of less than 1 (Figure 7), suggesting that all of the 13 PCGs had undergone purifying selection during their evolution in Papilionidae. The *COI* gene displayed the lowest Ka/Ks ratio (0.075), indicating strong purifying selection, a slower evolutionary rate, and greater conservation compared to other genes (Figure 7). In contrast, the highest Ka/Ks ratio observed in *ATP8* (0.236) suggests weaker selection pressure and potentially faster evolution (Figure 7).

### 3.4. Phylogenetic Relationships and Divergence Time

**Figure 8:**
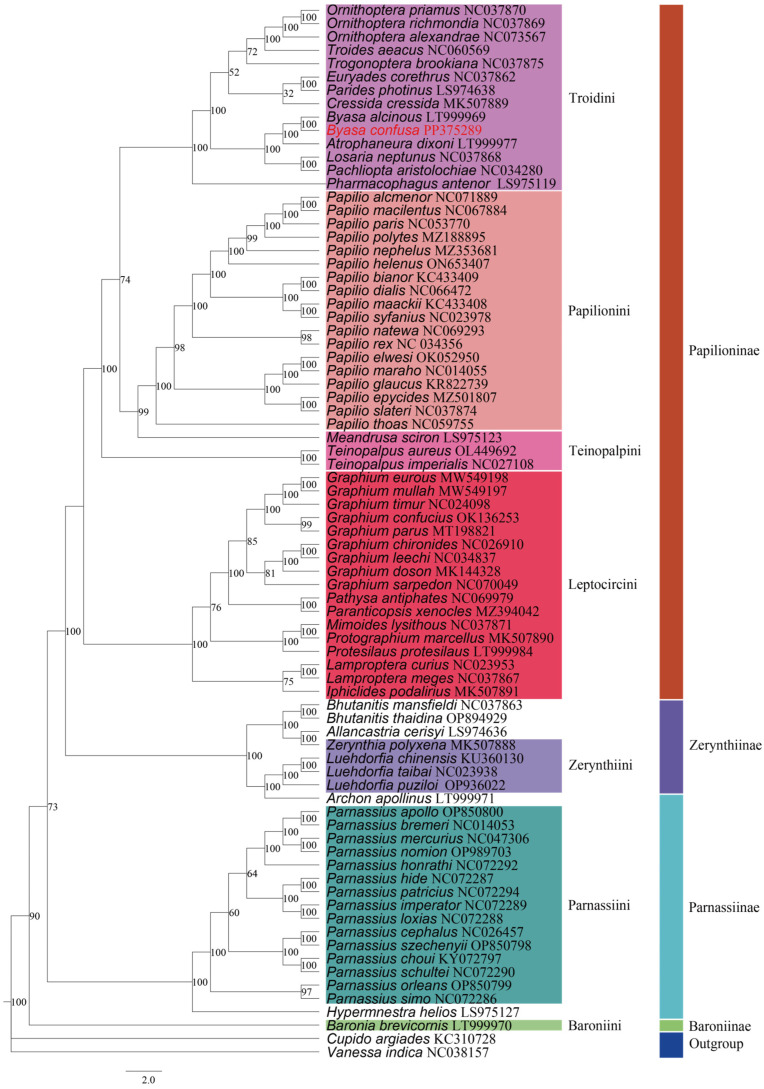
Maximum-likelihood (ML) tree based on 77 species of mitogenomes in the Papilionidae, with *Vanessa indica* and *Cupido argiades* as outgroup. Numbers on the nodes are bootstrap values based on 1000 replicates. The *B. confusa* is marked in red.

**Figure 9:**
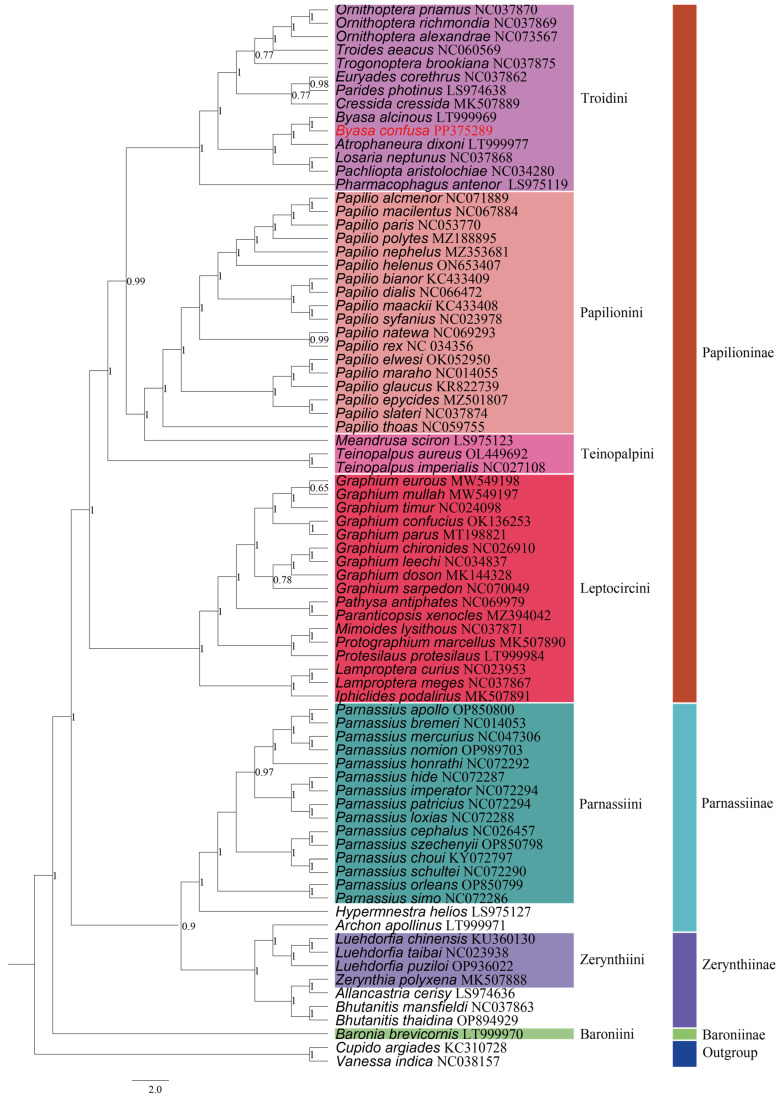
Reconstruction of a phylogenetic tree determined by Bayesian-inference methods based on 77 species of mitogenomes in the Papilionidae, with *Vanessa indica* and *Cupido argiades* as outgroup. Bayesian posterior probabilities (BPP) are shown at relevant branches of the BI tree. The *B. confusa* is marked in red.

To clarify the phylogenetic relationships among numerous significant subfamilies and tribes within Papilionidae, we constructed Bayesian-inference (BI) and maximum-likelihood (ML) phylogenetic trees using PCG123 and two rRNA datasets for 77 Papilionidae species, including *B. cofusa*, with *Vanessa indica* and *Cupido argiades* as outgroups. Our analyses yielded largely consistent tree topologies across the two concatenated datasets (Figure 8 and Figure 9). 

The trees revealed four distinct clusters corresponding to the subfamilies Papilioninae (PP = 1; BP = 100), Zerynthiinae (PP ≥ 0.9; BP = 100), Baroniinae (PP = 1; BP ≥ 90), and Parnassiinae (PP ≥ 0.9; BP ≥ 73). Within the four subfamilies, our ML analysis tree indicated a structure of (((Papilioninae + Zerynthiinae) + Parnassiinae) + Baroniinae), supporting that Papilioninae, Zerynthiinae, and Parnassiinae were all monophyletic. However, the subfamilies Zerynthiinae and Parnassiinae were paraphyletic in the BI tree. Notably, in both ML and BI analyses, the data uncontroversially supported the following relationship for Papilioninae at the tribe level: (((Troidini + Papilionini) + Teinopalpini) + Leptocircini). In the ML tree, both of the tribes Zerynthiini and Parnassiini exhibited good monophyly (BP = 100), which strongly supports the point of addressing these two taxa as separate subfamilies. However, in contradiction to the ML analysis, the two tribes may be sister groups to each other in the BI tree (PP ≤ 0.9), also implying that the two may belong to the same subfamily, i.e., Parnassiina. Additionally, the analyses confirmed the placement of *B. confusa* within the tribe Troidini of the Papilioninae subfamily.

**Figure 10:**
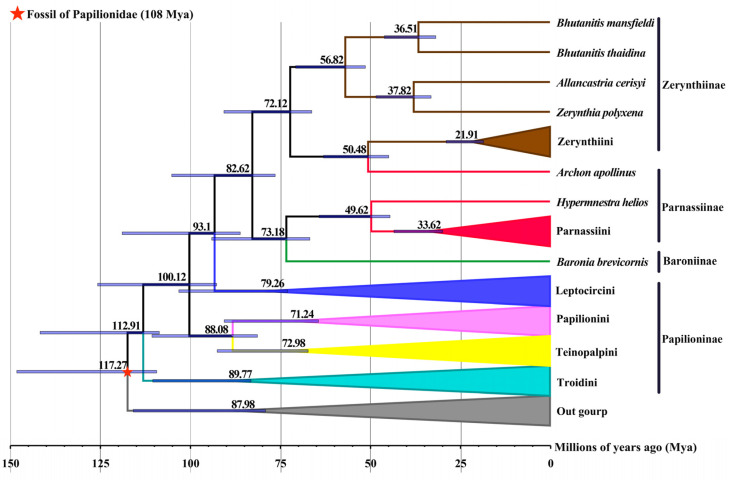
Estimated time tree of the Papilionidae. Blue bars indicate 95% posterior density intervals for node ages, while the red star represents the selected fossil calibration point.

Based on the BI tree of Papilionidae, the fossil calibration point of 108 Mya [57] between *Vanessa indica* and *Baronia brevicornis* was selected to estimate the divergence time (Figure 10). The subfamily Papilioninae was the earliest-appearing group in the investigation and was estimated to have diverged mostly during the Creataceous. Within the subfamily Papilioninae, the divergence between Troidini and the remaining lineages of Papilioninae occurred at around 112.9 Mya. Subsequently, the Troidini diverged at 89.8 Mya and the Leptocircini diverged at 79.3 Mya; while the Teinopalpini + Papilionini was derived at around 88.1 Mya, the two tribes began to diverge at around 73.0 Mya and 71.2 Mya, respectively. The remaining subfamilies of Papilionidae began to diverge at around 82.6 Mya (in the late Creataceous). Our results indicated that the evolutionary clades of the subfamily Parnassiinae formed a paraphyly in relation to both Baroniinae and Zerynthiinae, and all clades were derived at around 82.6 Mya. The subfamily Baroniinae appeared at around 73.2 Mya in the early Paleogene, and Zerynthiinae + Parnassiinae at around 72.1 Mya in the early Paleogene. Significantly, the tribes Parnassiini and Zerynthiini initiated divergence at roughly 33.6 Mya and 21.9 Mya, respectively.

## 4. Discussion 

### 4.1. General Characteristics

In the present study, we completed the sequencing of the mitochondrial genome of *B. confusa* for the first time and analyzed it in comparison with the 76 reported mitochondrial genomes of Papilionidae. The complete mitogenome of *B. confusa* showed a clear AT bias, and the order of the genes and the orientation of the open reading frames of the protein-coding genes were consistent with those of the other butterflies reported [8,9], and no gene rearrangements or deletions were found. Additionally, the overlapping region between *ATP8* and *ATP6* (7 bp) was present in the sequenced species, which is common throughout Lepidoptera [58,59].

Notably, our study reveals a consistent AT bias across all 77 species of Papilionidae analyzed, with AT content ranging from 77.2% to 82.2%. This bias is reflected in the relative synonymous codon usage of 13 PCGs and nucleotide composition. By comparing and analyzing the Ka/Ks values (all less than 1) of the 13 protein-coding genes (PCGs) within the known mitochondrial genomes of 77 butterfly species of Papilionidae, the result indicates that all 13 PCGs are under evolutionary purifying selection [60]. In particular, the *COI* exhibits the strongest purifying selection and high conservatism, as evidenced by its lowest Ka/Ks value (0.075). This further account strengthens the notion of the *COI* gene as an optimal molecular marker for taxonomic and evolutionary studies of butterflies [61].

### 4.2. Phylogenetic Relationships

During the process of investigating Papilionidae, we found that the classification of Zerynthiinae and Parnassiinae differs greatly among domestic and foreign studies [7,62,63], and some specialists consider Zerynthiini and Parnassiinii to be a sister group at the subfamily level [64,65]. Ding et al. made a preliminary discussion on the taxonomic status and genealogical relationship between the two based on morphology and geographic distribution, yet concluded that Zerynthiini should be classified as a tribe under the Parnassiinae and should not be regarded as a subfamily separately, but lacking molecular evidence [66]. Hauser and Caterino doubted the monophyly of Parnassiinae [67,68]; however, Omoto reconstructed the phylogeny of Parnassiinae using the *ND5* gene, which provided evidence for the monophyly of Parnassiinae [69]. In the present study, the results of the ML tree analysis indicate that both Zerynthiinae and Parnassiinae are monophyletic groups, supporting the subfamily status of Zerynthiinae, whereas the results in the BI tree differ; therefore, the present study is the initial exploration of a phylogenetic analysis for Papilionidae (including four subfamilies).

In previous investigations, as a larger subfamily in Papilionidae, there has been controversial phylogenetic relationships of tribes within Papilioninae. Some scholars abroad partitioned Papilioninae separately into three to five tribes [63,64,65,66,67], while Zhou and Wu classified Papilioninae into four tribes based on morphology, i.e., Troidini, Papilionini, Teinopalpini, and Leptocircini [7,62]. In this study, phylogenetic tree analysis supported the latter view by clustering Papilioninae into four monophyletic tribes at high confidence values.

However, there has been controversy over the phylogenetic status of Troidini. Aubert et al. phylogenetically analyzed partial sequences in 16S + *ND1* genes from representative species in each Papilionini genus, as well as from some other major Papilionidae taxa, indicating that Troidini is not a sister group to the Papilionini [70]. Using the *16S rRNA* gene as a genetic marker, Su et al. conducted a phylogeny study on Papilioninae that also found that Troidini was embedded within Papilionini, not showing better monophyly [71]; however, Zakharov et al. concluded that Troidini constituted a sister group with Papilionini through a comprehensive analysis of *COI* + *COII* + *EF-1α* sequences [72], which is in agreement with Miller and Simonsen [63,73]. In both the ML and BI trees of this study, Papilioninae shows a consensus evolutionary relationship: (((Troidini + Papilionini) + Teinopalpini) + Leptocircini). The results confirm that Troidini is a monophyletic group and also provide new molecular data for exploring the phylogenetic relationships of Papilioninae. However, further work and broader taxon sampling are necessary.

In summary, the contradictory patterns observed could be attributed to the still limited taxon sampling. Therefore, future studies should emphasize expanding the sample size and incorporating a wider range of molecular markers to obtain a more comprehensive understanding of the phylogenetic relationships among subfamilies and tribes within Papilionidae.

### 4.3. Divergence Time Estimates

Gaunt and Miles constructed a phylogenetic tree of holometamorphic insects by *COI* gene sequence analysis and estimated that Papilionidae diverged at around the late Cretaceous (89 May) [74]. Subsequently, Simonsen et al. and Nazari et al. dated the divergence of Papilionidae to 68 May and 90 May, respectively, based on biological morphology and molecular biology data [73,75], whereas Zakharov et al. delineated a divergence time range of 82.5 May to 89.1 May based on molecular biology data [72], all of which point to the Late Cretaceous. Our results advance the divergence of Papilionidae to around 112.9 May (Cretaceous period), and this estimate, although earlier than previous studies, is still within the confidence interval [72,73,74,75]. 

As we explored the divergence times of the tribes within Papilionidae, we found that the divergence time of Troidini was estimated by Braby et al. to be around 90 May by mitochondrial protein-coding genes, generally in agreement with the 89.8 May estimated in the present study [76]; meanwhile, the divergence time of Parnassiini (33.6 May) is similar to Nazari et al. and Michel et al.’s estimation (38 May to 34 May) [75,77]. Notably, the divergence time of Leptocircini (79.3 May) was remarkably earlier than that of Simonsen et al.’s estimate (44 May) [73], revealing that this tribe may possess an older evolutionary history. Such results broaden our understanding of the evolutionary history within Papilionidae and related tribes and also indicate the variation and complementarities in molecular dating across different research methods and datasets. 

## 5. Conclusions

In the present study, we offer comprehensive mitogenome data pertaining to *B. confusa*, engaging in a thorough analysis of its genetic structure and phylogenetic placement alongside other subfamilies and tribes. The mitochondrial genome of *B. confusa* spans 15,135 bp, displaying a notable bias towards AT composition. Our analysis underscores consistent trends in AT skew, codon utilization, single nucleotide polymorphisms, and sequence variation lengths across Papilionidae. Additionally, this paper provides a comprehensive summary of mitochondrial genomic attributes characterizing the Papilionidae family, serving as a valuable resource for future taxonomic studies.

The phylogenetic analysis conducted in this study delineated *B. confusa*’s placement within the *Byasa* genus, Troidini tribe, and Papilioninae subfamily, mirroring traditional morphological classification. According to the present comparative study, the status of Zerynthiinae as a distinct subfamily is preliminarily confirmed, especially notable in the ML analysis, whereas the monophyly of Papilioninae has further been supported, fulfilling the initial exploration of a phylogenetic analysis on Papilionidae (including four subfamilies). Furthermore, the controversial evolutionary relationships among tribes within Papilioninae are resolved.

As compared to other related studies, this study utilized a larger sequence set, resulting in a more convincing reconstruction of Papilionidae’s phylogenetic relationships than other related studies. However, there were also some shortcomings in this study, Including multiple discrepancies in the estimation of time divergence. In further research, more accurate estimates of divergence times are necessary with more precise fossil records for calibration and more complete sampling.

## Figures and Tables

**Table 2:** Positions and features of the genes in *B. confusa* mitogenome.

Gene	Strand	Genes Position	Size (bp)	Intergenic Spaces (bp) Number	Start-Codon	Stop-Codon
*tRNA^Met^*	J	1–63	63	0		
*tRNA^Ile^*	J	64–128	65	0		
*tRNA^Gln^*	N	126–194	69	−3		
*ND2*	J	228–1241	1014	33	ATT	TAA
*tRNA^Trp^*	J	1240–1304	65	−2		
*tRNA^Cys^*	N	1297–1362	66	−8		
*tRNA^Tyr^*	N	1363–1427	65	0		
*COI*	J	1430–2965	1536	2	CGA	TAA
*tRNA^Leu (UUR)^*	J	2961–3027	67	−5		
*COII*	J	3028–3709	682	0	ATG	T
*tRNA^Lys^*	J	3710–3779	70	0		
*tRNA^Asp^*	J	3780–3847	68	0		
*ATP8*	J	3848–4018	171	0	ATT	TAA
*ATP6*	J	4012–4689	678	−7	ATG	TAA
*CO III*	J	4689–5477	789	−1	ATG	TAA
*tRNA^Gly^*	J	5481–5545	65	3		
*ND3*	J	5546–5899	354	0	ATT	TAG
*tRNA^Ala^*	J	5898–5960	63	−2		
*tRNA^Arg^*	J	5961–6023	63	0		
*tRNA^Asn^*	J	6024–6088	65	0		
*tRNA^Ser (AGN)^*	J	6088–6148	61	−1		
*tRNA^Glu^*	J	6149–6216	68	0		
*tRNA^Phe^*	N	6215–6280	66	−2		
*ND5 (R)*	N	6286–8025	1740	5	ATT	TAA
*tRNA^His^*	N	8026–8089	64	0		
*ND4*	N	8089–9428	1340	−1	ATG	T
*ND4L*	N	9431–9721	291	2	ATG	TAA
*tRNA^Thr^*	J	9724–9788	65	2		
*tRNA^Pro^*	N	9789–9853	65	0		
*ND6*	J	9856–10,200	345	2	ATA	TAT
*CytB*	J	10,389–11,537	1149	188	ATG	TAA
*tRNA^Ser (UCN)^*	J	11,537–11,603	67	−1		
*ND1*	N	11,620–12,558	939	16	ATG	TAA
*tRNA^Leu (CUN)^*	N	12,560–12,629	70	1		
*16S rRNA*	N	12,605–13,963	1359	−25		
*tRNA^Val^*	N	13,965–14,028	64	1		
*12S rRNA*	N	14,029–14,832	804	0		
*CR*		14,833–15,135	303	0		

Note: J and N indicate the majority coding strand and the minority strand, respectively; T indicates the incomplete stop codon.

**Table 3:** Nucleotide composition of the *B. confusa* mitogenome.

Feature	Proportion of Nucleotides						
	A (%)	T (%)	C (%)	G (%)	A + T (%)	AT Skew	GC Skew
Whole genome	38.2	42.8	11.6	7.5	81	−0.06	−0.21
Protein-coding genes	33.2	46.3	10	10.5	79.5	−0.17	0.02
tRNA genes	41.3	40.4	7.8	10.5	81.7	0.01	0.15
16S rRNA	44.96	39.37	5.15	10.52	84.33	0.07	0.34
12S rRNA	45.52	39.55	5.1	9.83	85.07	0.07	0.32
Control region	43.89	51.16	3.63	1.32	95.05	−0.08	−0.47

## Data Availability

The genome sequence data that support the findings of this study are openly available in the GenBank of NCBI at (https://www.ncbi.nlm.nih.gov/) under accession no PP375289 on 21 February 2024. The associated BioProject, SRA, and Bio-Sample numbers are PRJNA1058706, SAMN39187394, and SRR27376806.

## Supplementary Materials

The following supporting information can be downloaded at: https://www.mdpi.com/article/10.3390/genes15070964/s1, Table S1: Nucleotide composition and skewness of 77 mitochondrial genomes of the family Papilionidae.
